# Mr. Bennett's Extraordinary Case of Abscess

**Published:** 1801-10

**Authors:** H. Leigh Thomas

**Affiliations:** Leicester Square


### To Dr. BATTY.

DEAR SIR,

I Have enclofed an extract of a letter which I received fome time ago from Mr. Bennett, an intelligent practitioner, at Shifnal, in Shropfhire, which I requeft you to infert in the Medical and Phyfical Journal. To render the cafe more compleat, I have fubjoined an accurate analyfis of the calculus, performed by my friend, Dr. Pearfon. The pin, with the remaining portion of the incruftation, is now in my poffeffion; its original length was fomewhat more than an inch, and about three-quarters in its circumference. It is not unufual to meet with needles, and other pointed fubftances, having paffed through different parts of the body, without fuffering any erofion erofion, or acquiring in their paffage any adventitious matter; probably the incruftation, in the prefent inftance, may have accumulated during the formation of the abfcefs, for we occafionally obferve a fimilar fubftance where ulceration has been produced by the long continued preffure of hard bodies, more particularly upon fecreting furfaces, as the vagina, &c.

I remain, dear Sir,
Your's very truly,
H. LEIGH THOMAS.

*Leicefter Square,*

*September* 18, 1801.

---

*An extraordinary Case of Abscess.* *By* Mr. Bennett.

Oct. 15, 1793, I was fent for to a collier's wife in the neighbourhood, who had lately lain-in of her firft child. She complained of violent pain in the right fide of the abdomen, which made me conclude that fome kind of inflammation was taking place. I ordered the part to be frequently fomented with a decoction of wormwood and camomile, applying after each time, fpt. camphorat. fprinkled upon warm flannel; alfo two table fpoons full of fperma ceti mixture, with fal. nitri to be taken every four hours. In the fpace of two days fhe was free from pain, but a tendernefs in the part remained on preffure, attended with a flight tumefaction, but not the leaft difcolouration whatever. The fomentation, &c. were difcontinued; the mixture taken regularly till the 26th, when a flight cough and expectoration coming on, and being much reduced in flefh and ftrength, her friends had an idea that fhe was going rapidly into a confumption; and, being in low circumftances, fignified to me that they could not afford any further medical attendance, as they thought it anfwered no purpofe. I therefore heard no more of her till June 24 following, when, to my great aftonifhment (fuppofing her dead long fince) I was again fent for to her, and found a confiderable abfcefs on the point of breaking, which had made its appearance rather fuddenly, without any great degree of pain, about half way between the pubes and umbilicus, and, in refpect of health, feemed confiderably better than when I laft faw her. I ordered a bread and milk poultice to the part, frequently renewed till the abfcefs burft, (fhe being averfe to having it opened with a lancet) which happened the next day, and difcharged a great quantity of thin whitifh pus. On the 27th, I faw her, and found the opening of a fufficient fize, and in the moft depending part, with no extenfive finus that I could difcover, or confinement of matter in any direction. I advifed it to be dreffed fuperficially with a mild digeftive ointment twice a day, and a poultice applied over the dreffing; alfo the moft nourifhing ing food ſhe could procure, and ſome wine, the diſcharge being very profuſe. Medicines ſhe would not take. I ſaw her a few times in the months of July and Auguſt, and conſidered her in a ſimilar way to one lingering under a Pſoas Abſceſs, ſhe being little more than a ſkeleton a ſecond time. I heard no more of her till November 11, when ſhe ſent for another box of digeſtive, and to inform me that a ſubſtance covering a pin (as they deſcribed it) with the point juſt viſible, had worked out of the abſceſs a few days before; that the diſcharge was much leſſened, of a thicker conſiſtence, and that ſhe began to feel herſelf better. In the courſe of the winter (the wound ſoon healing up) ſhe perfectly recovered; and towards the end of the year 1795, brought me the calculus, and, being pregnant, engaged me to attend her the following February, which unfortunately I was not able to perform, but learned ſhe had an eaſy labour.

It is difficult to ſay in what manner, or at what part, the pin was introduced into the body, and ſtill more difficult to ſpeak of its courſe. However, I cannot help thinking that it muſt have been puſhed in during a ſtrong labour pain; but whether in the part firſt affected, or where it came out, it is impoſſible to ſay.

---

### To Mr. THOMAS.

Dear Sir,

I am truly aſhamed to have been ſo long in your debt, from my promiſe to examine the calculous incruſtation on a pin lodged in the cellular membrane of the abdomen.

At length I have finiſhed a ſet of experiments with ſome care, and the reſult is, that the calculus conſiſts of above two-fifths of its weight of animal matter, as in bone, which are united to the remaining three-fifths of phoſphate of lime. There is certainly neither uric oxide, ſo commonly found in human urinary calculi and tophi, nor volatile alkali; but I am not ſure that there is not a ſmall proportion of magneſia alba. This reſult is very conſiſtent with ſome analyſes I have made of other calculi, partciularly of incruſtations on the teeth, improperly called *tartar*.

I am, dear Sir,

With ſincere regard,

Your's truly,

